# Phagocytosis of Particulate Antigens – All Roads Lead to Calcineurin/NFAT Signaling Pathway

**DOI:** 10.3389/fimmu.2013.00513

**Published:** 2014-01-09

**Authors:** Jan Fric, Teresa Zelante, Paola Ricciardi-Castagnoli

**Affiliations:** ^1^Singapore Immunology Network (SIgN), Agency for Science, Technology and Research (A*STAR), Biopolis, Singapore

**Keywords:** NFAT, dendritic cells, innate immunity, calcineurin, TLRs, phagocytosis, dectin-1 receptors, marco

Antigen-presenting cells (APC) possess multiple cell surface receptors that recognize common microbe-associated antigens as well as immune complexes and inert particles. Upon encountering such antigens these receptors must cooperate to achieve phagocytosis and trigger signaling cascades that initiate the innate immune response. While the stimuli initiating these signaling cascades are diverse, recent data have revealed that their effects in APC and particularly in dendritic cells (DC), all have something in common: downstream activation of nuclear factor of activated T cells (NFAT). NFAT is a family of transcription factors that has emerged as a key mediator of the initiation of immune responses by APCs, and specifically of IL-2 production by DC as reviewed (1). Intriguingly NFAT activation now seems to be the shared endpoint of several signaling pathways that all begin with uptake of particulate antigens.

Notably, the NFAT family members appeared at the origin of vertebrates whereas nearly all the other signaling pathways, including NFκB pathway, are very ancient and present in all invertebrate’s species. NFAT signaling plays essential roles in vertebrate organogenesis and development but also in the formation of adaptive immunity. In addition, G. R. Crabtree has suggested that “NFAT may have contributed to the evolutionary adaptation of innate immunity: e.g., minimize the costs of inflammation by collaboration with adaptive immunity” (2). It is likely that the ability of DC to link innate with adaptive immunity might be the result of DC’s ability to couple phagocytic functions to NFAT activation, leading to extensive gene reprograming.

This is the latest in a series of new hypothesis to better understand of the true complexity of the process of pathogen sensing, uptake, and response in APC and in particular in DC. But as is so often the case, with new hypotheses and knowledge has come new questions: how can such diverse stimuli all converge on similar pathways of immune activation? How do APCs integrate signaling from multiple immune uptake receptors? And how can we explain the difference in APC responses to soluble and particulate antigens?

In this article we will review the recent steps forward in our understanding of the intricate cross-talk between pathways of phagocytosis and immune signaling in APC, and the evidence that NFAT activation is a unique hallmark of this process.

## Phagocytic Receptors as Master Regulators of Uptake

Pattern recognition receptors (PRRs) are expressed abundantly by APCs, both on the cell surface and in intracellular compartments, and are ligated by conserved microbe-associated molecular patterns (MAMPs). Signaling by PRRs is important for innate immune cell activation, maturation, antigen processing, and presentation, and it now seems to be influenced by the process of phagocytosis itself. For example, it has long been known that responses to soluble and particulate forms of the same MAMP can vary enormously, but it is only recently that the interaction of phagocytosis and PRRs has been implicated in the mechanism of this distinction.

Phagocytosis and MAMP detection by APC have often been considered as complimentary but separate processes; however, it was noted that during microbe uptake, certain cell surface PRRs were actively recruited to the forming endosomes or phagosomes together with the microbial load. This subset of PRRs, including C-type lectin receptors and CD14, was termed the “Phagocytic Receptors”(3), and has since been extended to include opsonic receptors such as Fc receptors, which mediate uptake of immune complexes. However, it now seems that Phagocytic Receptors do not only mediate uptake of particulate antigens, but can also determine the recruitment, activation, and intracellular signaling of other PRRs during the process of phagocytosis and antigen/pathogen degradation (4).

## Phagocytic Receptors Co-Ordinate Complex Immune Responses to Simple MAMPs

Lipopolysaccharides (LPS) are highly immunogenic MAMPs on Gram-negative bacteria, and are recognized by several receptors on the APC surface; CD14, for example, is well-established as a non-opsonic Phagocytic Receptor for bacteria (5), but has recently been found to mediate the internalization of another LPS receptor, TLR4, via signaling through the tyrosine kinase Syk and PLCγ_2_ (6). What is particularly interesting about TLR4 is the effect that this process of internalization has upon the resulting signaling pathways that are triggered. Ligation of TLR4 by LPS at the plasma membrane activates the adaptor proteins TIRAP and MYD88, which enable pro-inflammatory gene transcription. However, once internalized with its ligand, TLR4 instead promotes TRAM-TRIF signaling, culminating in the production of type I IFNs (7, 8). Thus CD14 controls TLR4 internalization, and specific adaptor localization controls the downstream signaling pathways that result.

Alongside its interaction with TLR4, CD14 is also required for LPS signaling through Src-family kinases and PLC_γ2_ activation. As shown in murine DC this leads to Ca^2+^ flux and the activation of the phosphatase calcineurin, which causes NFAT nuclear translocation and so the release of IL-2 (9–11).

## Phagocytic Receptors Facilitate Distinction of Soluble and Particulate MAMPs

Whole microbes are significantly more potent immune stimulators than the soluble versions of their MAMPs, but understanding how APCs sense this difference has been challenging. Substantial progress was made recently when a study exposing murine DC to either soluble or bead-bound LPS revealed stronger triggering of Syk/PLC_γ2_ in response to “particulate” LPS (6), possibly resulting in increased Ca^2+^influx-dependent NFAT activation and transcription of downstream targets. This hinted at the importance of the NFAT pathway in determining immune outcomes to particulate antigens, however it was an elegant set of studies on the C-type lectin receptor, Dectin-1, that illustrated a molecular mechanism underlying APC detection of MAMP forms (12).

**Figure 1:**
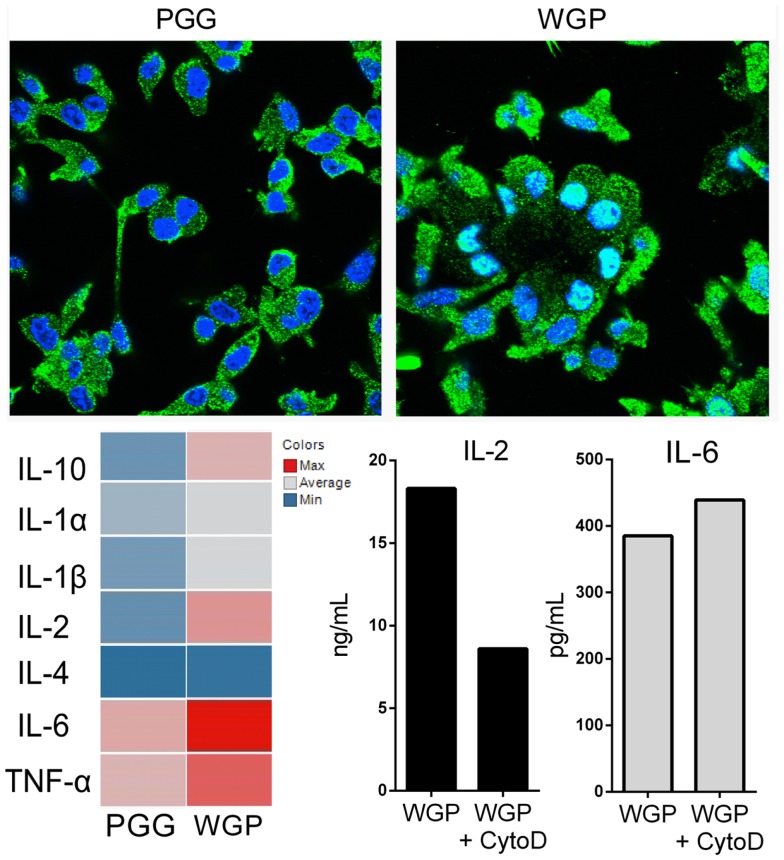
**Confocal microscopy of NFAT1 translocation in dendritic cell line (D1) upon treatment with 1,3-β glucan in both soluble form PGG (left) and aggregate of particles WGP (right)**. Both treatments lead to a different specialized cytokine pattern (bottom, left) measured by Luminex assay uniquely dependent on ligand solubility and indeed on the activation of mechanisms of endocytosis or frustrated phagocytosis. Blue, DAPI; Green, NFAT. The blocking of phagocytic process with Cytochalasin D results in inhibition of NFAT dependent cytokine (IL-2) while NFκB controlled response remain unchanged (IL-6), as detected in supernatants of D1 cells stimulated with particulate 1,3-β glucan (bottom, right).

Dectin-1 is a non-opsonic Phagocytic Receptor that recognizes the major component of the outer fungal cell wall, 1,3-β glucan (13–15). Upon ligand binding and receptor dimerization, the hemi-ITAM motif in the cytoplasmic tail of Dectin-1 is phosphorylated by Src-family kinases, which enables recruitment and activation of Syk. In turn, Syk mediates the MAPK response, canonical and non-canonical NFκB activation and also, importantly, stimulates the Ca^2+^ flux that drives calcineurin dephosphorylation of NFAT, leading to its nuclear translocation (15). This NFAT signaling is clearly linked with the particulate form of 1,3-β glucan and phagocytosis (Figure 1) and inducing different cytokine pattern than soluble form (Figure 1).

What has now emerged is that the differences in this process following ligation by soluble versus particulate ligands enable receptor-driven distinction between the two forms of the same antigen. The soluble β-1,3/β-1,6-linked glucans isolated from the cell wall of *Saccharomyces* bind single molecules of Dectin-1 and induce hemi-ITAM phosphorylation by Src-family kinases, but downstream signaling events, including calcium flux, are limited and transient. This results from expression of the membrane phosphatases CD45 and CD148 alongside Dectin-1, which serve to de-phosphorylate the Src kinases thereby impeding effective Dectin-1 signaling (12). In contrast, the aggregation of the β-1,3 glucan ligand on the surface of a yeast enables a parallel aggregation of Dectin-1 on the APC surface which increases receptor avidity and results in the formation of a “phagocytic synapse.” The close physical arrangement of ligands and receptors in the synapse excludes CD45 and CD148, releasing the brakes on sustained Src activation, and enabling induction of the cytoskeletal rearrangements required for phagocytosis, as well as the sustained calcium flux that is linked to NFAT activation and initiation of transcription of NFAT dependent cytokines (Figure 1).

## Phagocytic Receptors Deliver Extracellular MAMPs to Intracellular PRRs

The macrophage receptor with collagenous structure (MARCO) (16) is a non-opsonic multi-ligand Phagocytic Receptor whose function remained an enigma for many years; while MARCO is implicated in the pathogenesis of many inflammatory diseases, it is completely unable to initiate pro-inflammatory signaling itself (17). Several studies now indicate that it is MARCO’s interactions with other PRRs that define its role in shaping responses to particulate antigens. For example, strong and sustained signaling through the intracellular PRR TLR3 in response to the ligand PolyIC, required MARCO for rapid delivery and concentration of the ligand in the phagosome (18). In the case of the mycobacterial cell wall glycolipid trehalose 6,69-dimycolate, MARCO serves to enhance signaling trough the TLR2/CD14 complex (Bowdish PlosOne, 2009), where both molecules are known to be able to induce NFAT activation (10, 15). Indeed, MARCO-mediated phagocytosis of sterile particulates such as silica is the first step in a signaling cascade ending in NFAT activation and TNF transcription (19, 20). Cytoskeleton rearrangement has been observed as a result of MARCO clustering in activated DC (21).

## Phagocytic Receptors Link Antigen Uptake to PRR Activation

FcγRs are members of the immunoglobulin superfamily and are opsonic Phagocytic Receptors that recognize the Fc portion of antibodies bound to their cognate antigen. This process is central to the clearance of pathogens during infection, but has also been well studied for its role in autoimmune disease: in systemic lupus erythematosus (SLE) large self-DNA-containing immune complexes (DNA-ICs) are internalized by FcγRs on the surface of DC that then produce pro-inflammatory cytokines which contribute to disease pathology (22, 23). Signaling via the FcγR is integral to both these processes; DNA-IC binding to the FcγR induces its phagocytosis and also triggers activation of Src-family kinases, which first drive the cytoskeletal rearrangements needed to recruit the DNA-sensing TLR9 to the phagosome, and subsequently mediate phosphorylation of TLR9 (23). TLR9 activation in turn results in the recruitment and activation of the kinase Syk, and also activates the adaptor MYD88 which induces NFκB-mediated cytokine gene transcription and the secretion of type I IFNs. Whether FcγR-mediated uptake and immune activation specifically link with NFAT nuclear translocation has not yet been assessed, though the central role of activated Syk is suggestive (24). However, the related Phagocytic Receptor FcεRI (25), does trigger substantial calcium flux and NFAT translocation following ligand binding (26–28).

## Concluding Remarks

It is increasingly evident that the processes of immune uptake and immune signaling can no longer be considered as discrete, but rather are highly integrated by APC in order to induce multiple and inter-linked signaling pathways. One aspect of this is the roles of Phagocytic Receptors, some of which have been highlighted here. Despite diverse molecular structures and varied ligands, members of the Phagocytic Receptor family share the ability to direct downstream signaling toward triggering calcium influx, followed by calcineurin activation and NFAT translocation. NFAT signaling has been studied within myeloid cells (11, 29). Here we propose that the activation of calcineurin-NFAT signaling might be considered a hallmark of successful initiation of early innate responses toward phagocytosed particulate antigens. Interestingly, there is now evidence that NFAT activation may be part of a multi-component signature of the APC response to particulates; a recent study has shown that a common characteristic of the phagosomes formed during uptake by FcγRs, MARCO, and Dectin-1 is the accumulation of the autophagy marker LC3 (30). While the full implication of this observation remains to be investigated, the presence of LC3 implies a degree of cross-talk between pathways of particulate uptake and the non-canonical autophagic response of cells to ligands likely to include DNA-IC, Mycobacteria, and fungi.

These advances in understanding of the Phagocytic Receptors are already beginning to pay dividends; MARCO-mediated uptake of antigen-coated microparticles has successfully been exploited to ameliorate disease in a murine model of autoimmune encephalomyelitis (EAE) (31), while particles coated in the Dectin-1 ligands β-1,3-linked and β-1,6-linked glucans have been used as a potent vaccine delivery system in mice (32). Further understanding of the fine-tuned mechanisms underlying particulate uptake and immune signaling in APC, and the ensuing innate outcomes, e.g., the production of regulatory cytokines by DC such as IL-2 and IL-23 (1) has significant potential to improve our ability to design effective vaccines against infectious diseases and for the treatment of autoimmune conditions.

## Conflict of Interest Statement

The authors declare that the research was conducted in the absence of any commercial or financial relationships that could be construed as a potential conflict of interest.
